# Microsatellites Associated with Growth Performance and Analysis of Resistance to *Aeromonas hydrophila* in Tambaqui *Colossoma macropomum*

**DOI:** 10.3389/fgene.2018.00003

**Published:** 2018-01-18

**Authors:** Raquel B. Ariede, Milena V. Freitas, Milene E. Hata, Vito A. Mastrochirico-Filho, Fabiana Pilarski, Sergio R. Batlouni, Fábio Porto-Foresti, Diogo T. Hashimoto

**Affiliations:** ^1^Aquaculture Center of Unesp, São Paulo State University (Unesp), Jaboticabal, Brazil; ^2^School of Sciences, São Paulo State University (Unesp), Bauru, Brazil

**Keywords:** animal improvement, aquaculture, bacterial challenge, MAS, QTL

## Abstract

Tambaqui, *Colossoma macropomum*, is the main native fish species produced in Brazil, and is an important species for genetic improvement in aquaculture. In addition, breeding studies on this species can be optimized with the use of molecular markers associated with productive phenotypes. The objective of the present study was to test the performance of growth traits and resistance to the bacteria, *Aeromonas hydrophila*, in association with microsatellite markers in *C. macropomum*. In this study, three full-sib families were subjected to bacterial challenge and morphometric growth assessments. Tambaqui families subjected to the bacterial challenge differed significantly in death time and mortality rate. There was, however, no association between resistance to bacteria and microsatellite markers. In relation to growth traits, we observed a marker/phenotype association in two microsatellites. The marker in the 6b isoform x5 gene (*TNCRC6b*) was associated with length, whereas an anonymous marker was associated with height. The present study highlighted the evaluation of molecular markers associated with growth traits, and can serve as the basis for future marker-assisted selection (MAS) of tambaqui.

## Introduction

Tambaqui (*Colossoma macropomum*) is a freshwater fish belonging to the family Serrasalmidae, with natural occurrence in the Amazon and Orinoco basins (Jégu, 2003). Its body shape is similar to that of piranhas (round-shaped fish), reaching up to 1 m in length and a weight of up to 30 kg (Goulding and Carvalho, 1982). The natural food of tambaqui, as opposed to the carnivorous piranhas, is composed mainly of fruits and seeds (Lucas, 2008). Aquaculture production of tambaqui has increased over the past 10 years in several countries of South America, and particularly in Bolivia, Brazil, Colombia, Ecuador, Peru, and Venezuela (reviewed in Valladão et al., 2016). Tambaqui is currently considered the most important native fish produced in Brazilian aquaculture, with approximately 135 thousand tons produced in 2015 (IBGE, 2016). Production of tambaqui is also widespread throughout Brazil, particularly in the north, northeast, midwest, and southeast regions of the country (MPA, 2013).

In South America, most of the fish production by aquaculture is resulting from exotic species such as tilapia, *Oreochromis niloticus*, and salmon, *Salmo salar* (Valladão et al., 2016). However, different from tilapia and salmon, which has 20 and 13 breeding programs for genetic selection, tambaqui has few studies in order to select individuals of better performance for aquaculture (Gjedrem et al., 2012; Marcos et al., 2016). Tilapia is the main fish produced in Brazil (approximately 220 thousand tons per year) (IBGE, 2016), and most of this production is a result of the genetically improved farmed tilapia (GIFT) program, a genetic selection project developed over several generations (Ponzoni et al., 2011). Knowledge about growth performance and disease resistance in aquaculture is often derived from research using model fish, such as rainbow trout (*Oncorhynchus mykiss*), salmon, and tilapia (Gjedrem, 2000, 2010; LaFrentz et al., 2016; Shoemaker et al., 2017). Similar studies, however, are scarce for native species such as tambaqui. Any additional information on the genetic improvement of this species will contribute significantly toward increasing its productivity in aquaculture operations.

Growth performance (e.g., weight gain and morphometric parameters) is the only trait that has been studied for improving the breeding of tambaqui (Marcos et al., 2016; Mello et al., 2016). However, disease resistance is another important characteristic that can be evaluated for the genetic selection of this species. In South Brazil, where temperature variations during station changes are frequent, tambaquis suffer from outbreaks of disease, resulting in economic losses to the producers. The bacterium *Aeromonas hydrophila* is one the main opportunistic pathogens that causes disease in tambaqui (Valladão et al., 2016). This microorganism is often associated with hemorrhagic septicemia, fin erosion, and distention of the abdominal cavity, among other pathological conditions (Paniagua et al., 1990), and causes mass mortality in several fish species (Abdel-Tawwab et al., 2008; Sirimanapong et al., 2014).

Molecular markers associated with productive traits are being increasingly used to improve breeding programs (Houston et al., 2012; Yáñez et al., 2014). Marker-assisted selection (MAS) can increase the genetic gain of target species in approximately 25–50% of cases, compared to traditional selection techniques (Ma et al., 2014). The MAS approach associates genotypes with phenotypes to increase accuracy when predicting the genetic values of selection candidates (Yáñez et al., 2014). Moreover, MAS can be performed during the early life stages, and is indicated for traits such as disease resistance that are difficult to measure directly in selection candidates (Ma et al., 2014; Taylor, 2014). Therefore, numerous studies have been performed on fish species to identify, characterize, and validate molecular markers associated with different traits, including growth performance and disease resistance (Song et al., 2012; Rodríguez-Ramilo et al., 2013).

The objective of this study was to evaluate growth parameters and resistance to *A. hydrophila* in three full-sib families of tambaqui, *C. macropomum*. Moreover, we tested a set of microsatellites, including anonymous and gene-associated markers, to verify the association of tambaqui genotypes with its growth performance and resistance to *A. hydrophila*.

## Materials and methods

### Ethics statement

This study was approved by the Ethics Committee on Animal Use (CEUA number 18.764/16) of Faculdade de Ciências Agrárias e Veterinárias, UNESP, Campus Jaboticabal, SP, Brazil. Fish were anesthetized during the experiments with benzocaine (40 mg/L) and all efforts were made to minimize suffering.

### Family obtainment

Three full-sib families of tambaqui were obtained by mating three breeding pairs from the Aquaculture Center of UNESP, Campus Jaboticabal, SP, Brazil. Spawning was induced using carp pituitary extract that was dissolved in saline solution (0.9% NaCl) and applied in two dosages at an interval of 12 h (first and second dosage of 0.6 and 5.4 mg/kg, respectively), according to the protocol described by Pinheiro et al. (1988). After hatching in 20 L conical incubators over 15 days, approximately 150 larvae per family were transferred to 60 L tanks and maintained there until they reached approximately 3 g of body weight. Juveniles were identified by pit-tags placed posteriorly, and transferred to three different 750 L tanks and maintained until 6 months after hatching.

### Bacterial challenge

*Aeromonas hydrophila* was previously isolated from diseased *Colossoma macropomum* and stored in 30% glycerol at −80°C. This pathogenic strain was subsequently cultured in a nutrient tryptic soy agar (TSA), Vegitone (Sigma-Aldrich, India), for 24 h at 28°C. The colony was then transferred to nutrient tryptic soy broth (TSB) (Fluka, Sigma-Aldrich, India) and cultured for 24 h at 28°C. After growth, the culture broth was centrifuged at 3000 × g for 10 min at 4°C (Eppendorf Centrifuge 5810). The supernatant was discarded and the bacterial pellet was resuspended in sterile phosphate-buffered saline (PBS). This washing procedure was repeated twice. The optical density (OD) of the solution (previously determined for LD_50_ tests—lethal dosage in 50% of fish) was adjusted to 0.8 at 600 nm (CDCP, WHO, 2003) in a spectrophotometer (2100 Unico, Japan). A sample of 100 μl of this solution was removed from the inoculum (used for the fish challenge test) for conducting serial dilutions and plate counts in duplicate on TSA. A LD_50_ test was previously conducted on 30 individuals (10 from each family), using the following dosages: 10^6^, 10^7^, and 10^8^ CFU (colony forming units)/ml of *A. hydrophila*.

In the final experiment, 48 full-sib animals were used from each family (3 families × 48 fish = 144 fish). Each family was further separated into triplicate treatment groups of 12 fish (12 fish × 3 treatment replicates = 36 fish), plus an additional 12 fish were used for the control group. Replicate and control groups were composed of 36 individuals (i.e., 12 × 3 families). Each replicate was maintained in 750 L tanks, using a recirculation system fitted with mechanical and biological filters. Water temperature was maintained at 30 ± 1°C.

After adjustment of the LD_50_ (1 × 10^8^ CFU), infection was induced via intraperitoneal inoculation with a dose of 0.1 ml of bacteria per 10 g of fish body weight. Fish in the control group were inoculated with saline solution. The experiment was conducted over a period of 15 days, in order to evaluate resistance traits of mortality rate and the time of death. To evaluate the resistance among families, death time variance was performed using Tukey's test (*p* < 0.05) and the mortality rate was assessed by a chi-square test (*p* < 0.01).

### Growth traits

Six growth traits were measured in 96 individuals (32 fish from each family). Traits assessed included head length (HL), head height (HH), standard length (SL), height from the beginning of the dorsal fin to the beginning of the ventral fin (H1), height from the end of the dorsal fin to the beginning of the anal fin (H2), and body weight (BW). The five morphometric traits were measured in millimeters using the Image J/Fiji 1.46 software and BW was measured by a precision digital balance (accuracy of 0.01 g). After measurements, correlation of traits was estimated using the Pearson correlation (r). We did not compare growth performance among the families because each family grew in different environments (individual tanks).

### Evaluation of microsatellite markers

We used 13 gene-associated microsatellite markers in total (c5009, c5837, c4604, c3592, c3842, c4296, c2311, c3818, c841, c4706, C2647, c3843, c3905) and 4 anonymous markers (r1366, r912, r415, r3808) for evaluating associations with growth and resistance traits (Ariede et al., 2018). All microsatellites were previously tested for polymorphism in the progenitors. However, only heterozygous markers in at least one of the progenitors were selected for analysis in the progeny. Only nine microsatellites (c5837, c4604, c3592, c3842, c4296, c2311, c3818, r1366, r912) were selected with the previous characteristics, seven of which were gene-associated and two of which were anonymous markers. Individuals from family 1 were genotyped with the microsatellites c2311, c3592, c5837, c3818, and c4296; family 2 with c3842; and family 3 with c4604, c3842, r1366, and r912.

Genomic DNA was extracted from all full-sibs and parents, following the Wizard Genomic DNA Purification Kit (Promega) protocol. The sequencing strategy adopted in this study followed the protocols described by Schuelke (2010), and used the CAGtag primer (5′-CAGTCGGGCGTCATCA-3′) (Shirk et al., 2013) labeled with the fluorochromes HEX, FAM, or NED. The genotyping by polymerase chain reaction (PCR) was performed with the following reagents: 100 μM of dNTP, 1.5 mM MgCl_2_, 1 × Taq DNA buffer, 0.1 μM of each primer (F and R), 0.01 μM of CAGtag primer, 0.5 units of Taq Polymerase (Invitrogen), and 10–50 ng of genomic DNA. The cycling program for amplification consisted of: 9 cycles of 95°C for 30 s, 55–60°C for 30 s (adjusted for each primer set), 72°C for 20 s; followed by 30 cycles of 95°C for 30 s, 50°C for 30 s and 72°C for 20 s. During the first nine cycles, the annealing temperature of 55–60°C allowed for the incorporation of the primers (F and R) from the microsatellite loci. In the following 30 cycles, the temperature of 50°C facilitated the annealing of the fluorescent dye-labeled CAGtag primer. PCR products were analyzed by capillary electrophoresis in the equipment ABI3730 XL, using the DS-30 matrix with the GeneScan 500 ROX dye Size Standard (Thermo). We used the program GeneMapper 4.0 (Applied Biosystems) to analyze allele sizes.

Chi-square tests (*p* < 0.01) were performed on progeny genotypes to verify if they were distributed according to the Mendelian pattern. The association between microsatellite markers and growth traits and time of death was evaluated with the generalized linear model procedure of the analysis of variance (PROC-GLM ANOVA), using the statistical program, SAS (Statistical Analysis System, version 9.2). A linear animal model was used as follows: Y = m + G + e, where **Y** is the observed value of a given continuous characteristic (growth traits or time of death); **m** is the overall mean of the characteristic; **G** is the effect of the genotype; and **e** the random error effect (*p* < 0.05). The statistical model was based on that described by Ma et al. (2014), with some modifications. The mortality rate was considered as a binary variable (live = 1 and dead = 0); thus, this characteristic was evaluated by means of a contingency table (chi-square test, *p* < 0.01).

## Results

### Analysis of resistance to *A. hydrophila*

**Figure 1:**
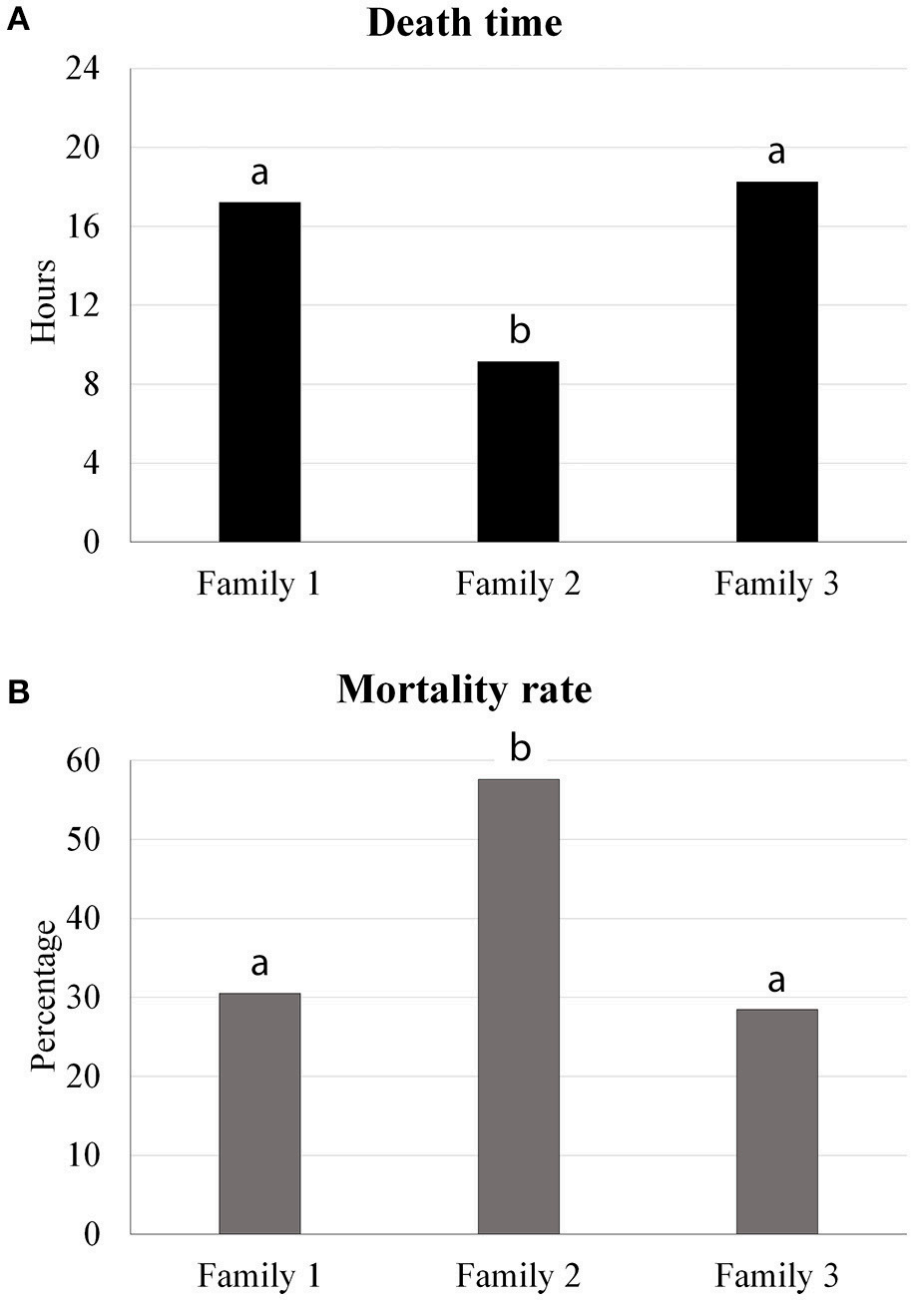
Traits evaluated in the bacterial challenge (death time and mortality rate) indicating significant differences between the tambaqui families.

All infected animals presented clinical signs of *A. hydrophila* infection. The average of death time observed in families 1, 2, and 3 was 17.20, 9.13, and 18.25 h, respectively, indicating significant differences between families 1 and 2, and families 2 and 3 (Figure 1A). Moreover, mortality rates were significantly different between the families (Figure 1B); the mortality rate of family 2 (57.6%) was higher than the mortality rates of family 1 (30.5%) and family 3 (28.5%). There was no fish mortality observed in the control treatment.

### Analysis of growth performance

The average BW in families 1, 2, and 3 was 44.22, 38.22, and 30.68 g, respectively. Body weights in families 1, 2, and 3 ranged between 12–70 g, 16–67 g, and 21–57 g, respectively. The average SL in families 1, 2, and 3 was 106.47, 103.69, and 96.01 mm, respectively. Other growth traits are described in Table 1. Phenotypic correlation indicated that all characteristics were significantly correlated (*p* < 0.01). Measurements of SL and H1, however, were poorly correlated with a correlation value of 0.37 (Table 2).

**Table 1:** Analysis of growth performance in three families of tambaqui *Colossoma macropomum*, including average values and standard deviation for weight and morphometric measures.

**Family**	**Traits**					
	**BW**	**HL**	**HH**	**SL**	**H1**	**H2**
1	44.5 ± 12.7	38.0 ± 4.9	34.5 ± 4.0	105.6 ± 11.3	55.4 ± 5.8	32.6 ± 4.7
2	39.8 ± 8.0	38.5 ± 4.8	35.2 ± 3.9	104.3 ± 18.0	55.6 ± 10.1	32.2 ± 4.1
3	31.7 ± 10.7	35.4 ± 4.6	31.1 ± 3.4	96.4 ± 8.2	47.4 ± 4.4	28.5 ± 3.0

*BW, body weight (g); HL, head length (mm); HH, head height (mm); SL, standard length (mm); H1, height from the beginning of the dorsal fin to the beginning of the ventral fin (mm); H2, height from the end of the dorsal fin to the beginning of the anal fin (mm)*.

**Table 2:** Analysis of Pearson correlation (r) between growth traits (weight and five morphometric measures) in three families of tambaqui *Colossoma macropomum*.

	**BW**	**HL**	**HH**	**SL**	**H1**
HL	0.71				
HH	0.70	0.94			
SL	0.73	0.84	0.83		
H1	0.62	0.58	0.62	0.37	
H2	0.73	0.66	0.70	0.73	0.68

*All values represented significant correlations (p < 0.05). BW, body weight (g); HL, head length (mm); HH, head height (mm); SL, standard length (mm); H1, height from the beginning of the dorsal fin to the beginning of the ventral fin (mm); H2, height from the end of the dorsal fin to the beginning of the anal fin (mm)*.

### Analysis of association with microsatellite loci

Genotypes of the parents with their respective microsatellites are described in Table 3. The markers c4604, c3842, and r1366 that were applied in family 3 did not follow the Mendelian segregation pattern (*p* < 0.01), and were therefore excluded from the association analysis. In relation to the analysis of resistance to *A. hydrophila*, significant association was not detected between microsatellites and death time or mortality rate. In contrast, growth traits had significant association with markers c3842 and r912 in families 2 and 3, respectively. Progeny genotypes and growth traits averages are described in Table 4. The gene-associated marker c3842 showed an association with SL, and the anonymous marker r912 was associated with H2. For the marker c3842, individuals with the genotype 183/183 had an average SL of 111.56 mm, whereas those with the genotype 183/186 showed an average SL of 98.33 mm (Table 4). Moreover, for the marker r912, fish with the genotypes 175/175 and 175/185 demonstrated an average H2 of 27.44 and 29.65 mm, respectively.

**Table 3:** Characterization of heterozygous microsatellites in at least one of the progenitors in three families of tambaqui *Colossoma macropomum*.

**Family**	***Loci***	**Genotype♂**	**Genotype♀**
1	c5837	174/179	174/174
	c2311	163/169	166/175
	c3818	204/214	204/210
	c3592	166/170	170/170
	c4296	118/122	118/118
2	c3842	183/186	183/183
3	c4604	142/151	148/151
	c3842	183/186	183/186
	r1366	261/277	261/277
	r912	175/175	175/185

*Genotypes are represented by allele size in base pairs (bp)*.

**Table 4:** Analysis of the microsatellite loci in the progeny and average values of growth traits (weight and five morphometric measures) by clustering individuals according to the respective genotype in three families of tambaqui *Colossoma macropomum*.

**Family**	***Loci***	**Progeny genotype**	**Genotype frequency**	**BW**	**HL**	**HH**	**SL**	**H1**	**H2**
1	c5837	174/174	0.34	42.55	37.61	34.61	104.80	53.60	34.15
		174/179	0.66	42.52	38.17	34.44	106.07	53.27	31.92
	c2311	163/166	0.25	36.50	36.07	32.96	101.72	51.14	30.39
		163/175	0.40	45.08	38.94	35.45	107.49	54.44	33.92
		166/169	0.19	41.67	37.26	33.49	105.26	53.37	32.32
		169/175	0.16	46.60	39.37	35.67	107.51	54.25	33.61
	c3818	204/204	0.24	39.29	37.61	34.01	102.54	51.52	30.41
		204/210	0.34	37.40	36.74	33.67	101.44	50.86	32.17
		204/214	0.18	43.75	36.88	34.10	106.19	54.52	32.77
		210/214	0.24	51.71	41.36	36.62	114.07	57.56	35.60
	c3592	166/170	0.59	42.47	37.28	34.18	104.84	53.06	32.24
		170/170	0.41	42.62	38.99	34.96	106.78	53.85	33.34
	c4296	118/118	0.62	43.60	38.30	34.83	106.20	53.49	32.60
		118/122	0.38	40.75	37.45	33.94	104.68	53.21	32.82
2	c3842	183/183	0.47	45.29	39.94	36.18	111.56	55.15	32.71
		183/186	0.53	36.50	38.13	34.81	98.33	54.42	32.38
3	c4604^*^	142/148	0.47	32.53	35.05	30.75	97.50	48.38	29.69
		142/151	0.22	30.43	34.67	31.30	95.06	46.04	27.09
		148/151	0.06	24.50	34.07	28.40	91.31	44.29	26.92
		151/151	0.25	32.88	36.92	32.22	96.39	47.16	27.81
	c3842^*^	183/183	0.03	34.00	35.74	30.19	86.76	42.95	24.28
		183/186	0.56	30.72	35.60	31.41	96.13	47.79	28.94
		186/186	0.41	32.77	35.03	30.72	97.29	46.98	28.17
	r1366^*^	261/261	0.06	29.00	32.21	29.85	94.11	46.71	27.93
		261/277	0.66	32.71	36.30	31.41	97.30	47.77	28.84
		277/277	0.28	29.78	33.91	30.62	94.47	46.35	27.76
	r912	175/175	0.53	30.35	34.18	31.02	94.41	46.29	27.44
		175/185	0.47	33.13	36.72	31.16	98.46	48.46	29.65

*Genotypes are represented by allele size in base pairs (bp). BW, body weight (g); HL, head length (mm); HH, head height (mm); SL, standard length (mm); H1, height from the beginning of the dorsal fin to the beginning of the ventral fin (mm); H2, height from the end of the dorsal fin to the beginning of the anal fin (mm)*.

* *Markers that did not follow the Mendelian segregation pattern (p < 0.01)*.

## Discussion

Challenge experiments of *A. hydrophila* in this study revealed significant variations in death times and mortality rates between the three families of tambaqui. Our results indicated that family 2 was more susceptible to *A. hydrophila* than families 1 and 3; death time was lower and the mortality rate higher in family 2, compared with those observed in the other two families. These results indicate genetic variation for this trait in the families of tambaqui analyzed in this study; selective breeding could thus be applied for resistance to *A. hydrophila* in tambaqui, as has been done in other model aquaculture species such as salmon, tilapia and rainbow trout (Silverstein et al., 2009; Ødegård et al., 2011; Wiens et al., 2013; Evenhuis et al., 2015; LaFrentz et al., 2016; Shoemaker et al., 2017). For example, Nile tilapia families challenged with the pathogen *Streptococcus iniae* showed an additive genetic component to resistance, indicated by the variation in survival rate; genetic selection methods can be thus applied in the production of animals resistant to this pathogen (LaFrentz et al., 2016).

Standard methods for disease prevention and control in the aquaculture industry include the use of vaccines, antibiotics, and management strategies (fish density, water flow, oxygen control, etc.), all of which are only partially effective. Vaccination is generally expensive and impractical for large-scale fish production, as it requires that all fish are individually treated (Yáñez et al., 2014). The indiscriminate use of antibiotics can lead to the development of populations of antibiotic-resistant bacteria, and pose a risk to human health and the environment (Vivekanandhan et al., 2002; Taylor et al., 2011). Conversely, selective breeding for disease resistance is a more effective, sustainable, and low-cost approach to improving fish health and performance, without the risks and inefficiencies posed by the other standard methods (Stear et al., 2001; Bishop, 2010).

This study did not identify the association of molecular markers with resistance to *A. hydrophila* in tambaqui. In general, as this trait is usually controlled by polygenic architecture, studies to search for association with resistance to pathogens have previously been conducted using hundreds of molecular markers on species such as turbot (*Scophthalmus maximus*), rainbow trout, salmon, Nile tilapia, and cod (*Gadus morhua*) (Pardo et al., 2008; Ødegård et al., 2010; Yáñez et al., 2014; Evenhuis et al., 2015; LaFrentz et al., 2016). Therefore, studies specifically on tambaqui are required to search for quantitative trait loci (QTL) of resistance to *A. hydrophila*, using a higher marker density.

The results of phenotypic correlation between morphometric measures in this study were similar to those previously reported for tambaqui by Mello et al. (2016); these authors demonstrated that selection for filet weight can be made on the basis of morphometric measures due to positive correlation between these characteristics. In both studies, weight had a higher phenotypic correlation with standard length and head length (*r* = 0.70 in both studies). Moreover, the highest phenotypic correlation was observed between head length and standard length (*r* = 0.84 in the present study, and 0.90 in Mello et al., 2016). This pattern of high correlation between body measurements and weight (*r* = 0.90) was also observed in Nile tilapia (Rutten et al., 2005), indicating that the selection for a specific trait would also result in the gain of other traits, as is the case for tambaqui.

The morphometric data observed in *C. macropomum* indicates that this species shows a large head length, which is not attractive for the commercial sector; a larger head length lowers the filet yield (Mello et al., 2016). As observed in both studies, there is a positive correlation between head length and weight; therefore, it is not possible to select individuals of greater body weight without them also having a larger head length.

Several studies using growth markers were performed on genes that are directly linked to this phenotype, such as the growth hormone (*GH*) gene and the insulin gene (*IGF1*) (Yue and Orban, 2002; Tsai et al., 2014). In a study with two tilapia species, six microsatellites were detected in four genes that were involved in growth and reproduction processes (Yue and Orban, 2002). The c3842 marker, identified in this study in association with growth, is inserted in the region of the repeat-containing 6b isoform x5 gene (*TNCRC6b*), located in the 5'-UTR region. This gene has already been described in mammals (humans and rats) and in fish (*Maylandia zebra*), and its function may be related to various biological processes such as the silencing of genes guided by micro RNAs, and the regulation of positive messenger RNA, among others. It is thus necessary to increase our understanding of the role of this gene and the possible relation between its molecular functions and differential growth in tambaqui. The results obtained from the association analysis with the gene-associated marker c3842 suggest that the allele 183, when homozygous, exerts influence on the increase of the mean SL of the animal.

The second microsatellite that showed association with growth was the anonymous marker r912. In general, anonymous markers are often used in QTL identification studies, because such markers are physically close to these loci and therefore closely linked (Reid et al., 2005; Bouza et al., 2012). Our results suggest that r912 is related to a second morphometric characteristic (Height 2). Moreover, and contrary to that observed in the gene-associated marker, increased height occurs when the r912 marker is heterozygous, suggesting that the presence of allele 185 exerts influence on animal height. To date, the reference genome of tambaqui is not available; therefore, it is not possible to characterize the loci of the r912 marker and the putative linked gene. A similar strategy of gene prospection was carried out in salmon, whose reference genome is sequenced, allowing the prediction of two markers in association with the genes *MEP1A* (meprin A subunit beta-like) and *PCNT* (pericentrin) (Houston et al., 2014; Tsai et al., 2016).

Currently, 100% of the tambaqui production is still based on stocks without genetic improvement. The identification of two markers (c3842 and r912) associated with growth in tambaqui will support and accelerate the processes for selection of superior genotypes using MAS. A classic example of QTL identification is the detection of a haplotype that is associated with approximately 80% of the genetic variation in salmon resistant to pancreatic necrosis infection (Houston et al., 2008, 2012). Owing to their efficacy, these markers are currently being successfully incorporated into breeding programs of Atlantic salmon in Norway and Scotland (Moen et al., 2009; Houston et al., 2010). The results of this study will thus likely increase our knowledge base of tambaqui aquaculture, and facilitate the development of future tambaqui breeding programs.

## Conclusion

This study provides new information on tambaqui (*C. macropomum*) growth traits to guide future breeding programs for this species. The prospective microsatellites highlighted in this study could be used in the validation of MAS in families of *C. macropomum*. This study also contributes to the development of a bacterial challenge protocol with *Aeromonas hydrophila* in order to characterize pathogen-resistant tambaqui families for selective breeding.

## Author contributions

RA–Acquisition, analysis and interpretation of data, draft of the work, development of intellectual content, writing of the manuscript, final approval of the version. MF, VM-F, FP, and SB–Draft of the work, development of intellectual content, final approval of the version. MH–Analysis and interpretation of data, draft of the work, final approval of the version. FP-F–Acquisition, Interpretation of data, development of intellectual content, final approval of the version. DH–Draft of the work, development of intellectual content, writing of the manuscript, final approval of the version.

### Conflict of interest statement

The authors declare that the research was conducted in the absence of any commercial or financial relationships that could be construed as a potential conflict of interest.
